# The Etiology of Neuralgia*Remarks made before the Chicago Medical Society, March 18, 1889.

**Published:** 1889-04

**Authors:** Henry M. Lyman

**Affiliations:** Professor of Physiology and Nervous Diseases, Rush Medical College; 533 West Adams Street


CHICAGO MEDICAL

JOURNAL AND EXAMINER.

VOLUME LVIII. APRIL, 1889. NUMBER 4.

### *THE ETIOLOGY OF NEURALGIA.**

BY HENRY M. LYMAN, M.D.

PROFESSOR OF PHYSIOLOGY AND NERVOUS DISEASES, RUSH MEDICAL COLLEGE.

I had hoped there would be a paper preceding my remarks in order to make clear what neuralgia is; for we shall find in discussing and studying the subject that it is rather a difficult thing in many instances to decide whether a given case of pain is to be considered as neuralgia, or as the result of an inflammation, deterioration, degeneration, or other change of a morbid character involving a nerve or nerve center. As there has been nothing said upon that point, I will take it for granted that the matter is understood, and that in speaking of neuralgias it is intended to speak of those pains that are sufficiently localized to be identified with nerve trunks, branches and terminals, and that are not produced by severe injuries, inflammations, or marked degenerations of a palpable character involving the nerve trunks or organs themselves. With that premise, I will speak of its etiology.

I would say in this connection that neuralgia is not a disease, but is rather a symptom of disease. We shall see that it may be caused by so many different conditions and lesions that it cannot be considered as a specific disorder. It must rather be considered as an evidence of disease involving the nervous system.

Now, when we consider the causes of neuralgia we shall find that they fall into several clearly-marked categories, such as used to be spoken of as predisposing causes and exciting causes, but it is rather difficult to draw a line between them, and therefore in my classification I shall consider as nearly as possible the actual causes of pain itself. In doing that we shall find, in the first place, that there are certain conditions which seem to predispose to its occurrence, that is, conditions of the body or of the general organization; and first among the prdisposing conditions may be mentioned heredity.

*Heredity* is a very general term. It is made to cover that assemblage of characteristics derived from our ancestors; and we find that the physiological and pathological peculiarities of the ancestral stock have great influence over the diseases of the descendants. Persons who are subject to neuralgia are very frequently found to be the offspring of parents who have experience of disease in their own persons, and have been weakened in such a way as to weaken their offspring. We find a familiar illustration of this in the children of persons who are tubercular or scrofulous, who are the victims of nervous dis-

*Remarks made before the Chicago Medical Society, March 18, 1889.

eases, who have been addicted to alcoholic excesses. The children of such parents are more liable to neuralgia than the children of those who have been more favored in circumstances and mode of life. We shall see presently why this is so.

*Age.*—Another of these predisposing conditions is said to be age. It is observed that neuralgia occurs more frequently at certain periods of life than at others. It is more frequent among children during the period of rapid growth. We do not find it among very young children. It is then an uncommon thing, yet it does sometimes occur, but more particularly as they approach the period of puberty. As they pass through the period of second dentition they are more liable to neuralgia than younger children. As individuals pass through the stage of middle life, 20 to 40, they seem to be more liable still to the disorder. This is due to the fact that during these years of life there is a larger number of persons who are subjected to the causes that more immediately operate to produce it. As we pass that period of life—the 40th year—the number of persons who exceed that age diminishes rapidly in consequence of the fact that so many die, and the number who are left to experience neuralgia diminishes in proportion. After the period of decline is reached, we find there is a tendency to neuralgias of certain kinds that are dependent upon deterioration of the nerve centers, and it is to causes of that description that the majority of neuralgias which occur later in life are to be ascribed.

*Sex* has been thought to be one of the predisposing conditions. It is true, neuralgia occurs with great frequency among females, but that is due to the fact that a great many females suffer from conditions of debility and exhaustion, and are the victims of certain diseases that also favor the production of neuralgia. The other sex also are victims, not perhaps from the same causes, but the number of cases is sufficient to lead us to believe that the female sex has no great monopoly in neuralgia.

*Anæmia* is another fruitful predisposing condition of neuralgia. It is to the existence of anæmia among the female sex that we must often ascribe the supposed predisposition of women. Starvation may be reckoned along with that condition. We find many persons suffering from unwholesome nutrition; the tissues are not properly nourished, and as a consequence of this imperfect nutrition of the tissues they become unstable in their organization, and more readily react to external influences. That reaction, if excessive, unusual or unnatural, will produce pain. We call it neuralgia. And among the other conditions that are allied to, and probably based in great part upon, anæmia, and upon an imperfectly nourished condition of the tissues, may be mentioned hysteria, neurasthenia, hypochondria. These, generally, constitute the conditions which are observed to predispose to the occurrence of painful affections of this character.

*Exciting Causes.*—We find these may be considered in two principal classes. Those that are molecular in their origin, consequent upon disturbances in the molecular constitution of the body; and a still larger class, perhaps, that are produced by direct intoxication of the tissues of the body, including the nervous tissues. Among the disturbances of molecular motion within the body, which produce either excessive, deficient or perverted modes of action may be reckoned cerebral commotion. This may have its origin in anything that produces over-excitement or agitation of the brain, as in the case of over-action of the sensory organs to powerful light, sounds, odors, tastes. All those reactions of the sense organs which are carried to excess may produce painful conditions of the nerves, which should be reckoned, strictly speaking, among the neuralgias. The same thing is true of commotions that originate under the activity of the intellectual organs of the brain. A person who works too hard, gets too little sleep; who uses his eyes at night reading or studying, if he has passed the period of childhood, is very liable to suffer from neuralgic pain that localizes itself in the back of the head and neck, and is largely due to over-excitement of the brain and to lack of rest.

Then, again, over-agitation of the emotional organs of the brain will produce similar results. Electrical excitement of the nervous apparatus, occurring either as a consequence of electrical disturbances of the atmosphere, or as a consequence of medical abuse of electricity, may produce neuralgic pains. Perversions of the circulation, by their effect upon the molecular movements of the brain and of the nerve centers, may produce neuralgic pains, many of which are supposed to be due to errors of circulation, either excessive or deficient, involving the spinal cord, the sensitive grey matter of the nerve centers in the spinal cord, or of the brain itself which takes cognizance of the impressions made upon the sense organs of the body. We find the action of heat and cold, if carried to excess, productive of painful states of the nervous system. You are all familiar with the simpler forms of neuralgia produced by the action of cold upon the extremities of the body in winter when the weather is cold; heat also will produce similar or analogous effects, if the person is exposed to heat of any kind for too long a time.

We shall find, too, that intoxication of the nervous system with inorganic poisons is a fruitful source of pain. I merely mention in passing the familiar effects produced by the introduction of lead, copper, mercury, arsenic, carbon disulphide, carbon monoxide, compressed air, and substances of that class into the body. We may say that all the metals or any of the chemical elements introduced into the body in excess may become a source of pain.

You are also familiar with the effects of metallic poisons of all descriptions. The gases also that belong to the inorganic kingdom, if they are introduced in excess into the body, may produce pains that are severe.

Then, we find that there is a class of intoxications in which the poisons that are derived by chemical process from the products of fermentation are capable of producing pain. Examples of these poisons are found in alcohol, chloroform, and ether. These substances are narcotic themselves. Several interesting cases have been reported of severe neuralgic pain following the administration of ether for surgical purposes. After recovery from the effects of the ether the patients for weeks and months have suffered with severe neuralgic pains. I mention these more common substances. All of the derivatives of fermentation are analogous to these, and, like alcohol, ether and chloroform, will be found in certain instances to be productive of painful conditions of the nerves.

Again, we find neuralgia may be produced by intoxication of the nervous system with vegetable poisons of the ordinary type, such as muscarine, opium, arnica, hydrocyanic acid, the solanaceous alkaloids, caffein, thein, nux vomica, and many other kindred substances. Here we must pause to recognize the fact that there are two ways in which the nerve centers can be acted upon in a painful way. One is through the direct action of the poison upon the nervous tissue, and the other (usually observed in cases of chronic intoxication) is consequent upon the modifications of nutrition and the deteriorations of structure which follow these modifications of nutrition. It may sometimes be an open question for debate as to which of these two actions is the real cause of the pain in an individual case. This we shall see illustrated still further in cases of intoxication with poisons of parasitic origin, as, for example, where we have to deal with the essential poisons of the fevers, such as typhoid fever, etc. At the outset of the fever the patient experiences intense pain, which is spoken of as headache, backache, aching in the limbs and in different parts of the body. This is due to the direct action of the specific poison upon the nervous organization of the body; then after the specific poison has run its course and has ceased to be primarily operative, we have disordered health, anæmia, semi-starvation, hysteria, and perhaps other conditions acting as secondary conditions that predispose to neuralgia. So that persons who are intoxicated in this way may suffer in a twofold manner from the consequences of the specific poisons.

It is occasionally observed that patients suffering with tubercular infection become the victims of intense neuralgic pains, which are supposed to be due to the action of the poison that is secreted or produced by the specific parasite. The same thing is true of syphilis and of gonorrhœa, after which may sometimes be observed that curious form of neuralgia, characterized by fugitive pains that persecute their victim for many months after the original infection has taken place.

Again, the poisons that produce septicæmia, pyæmia, and malarial infection, when introduced into the body, in like manner with the poisons that produce specific fevers, cause pain as one of their primary effects. As a consequence of alteration of the liquids and solids of the body through impaired nutrition thus occasioned, painful conditions may be perpetuated after the original poison has ceased to operate. In addition to the above must be mentioned the poisons derived from intestinal bacteria which exist in the stomach and intestinal canal and which, in conditions of ill health, produce large quantities of poisonous matter that is absorbed and circulated through the body operating upon the nervous apparatus, and producing a great deal of distress.

Lastly, we find that there is a class of neuralgias dependent upon intoxication with poisons of auto-genetic origin—poisons produced by the morbid action of the cell elements of the bodily tissues themselves, such as occur in rheumatism, gout, uræmia, excess of sugar, matters concerning which the present state of our knowledge is very imperfect. Such, then, is the account I would give of the causes of neuralgia and their classification.

533 West Adams Street.

